# Evaluation of an Intergenerational and Technological Intervention for Loneliness: Protocol for a Feasibility Randomized Controlled Trial

**DOI:** 10.2196/23767

**Published:** 2021-02-17

**Authors:** Peter Hoang, Colin Whaley, Karen Thompson, Venus Ho, Uzma Rehman, Karla Boluk, Kelly A Grindrod

**Affiliations:** 1 Department of Medicine Cumming School of Medicine University of Calgary Calgary, AB Canada; 2 enTECH Computer Club University of Waterloo Waterloo, ON Canada; 3 School of Pharmacy University of Waterloo Waterloo, ON Canada; 4 Michael G DeGroote School of Medicine McMaster University Hamilton, ON Canada; 5 Department of Recreation and Leisure Studies University of Waterloo Waterloo, ON Canada; 6 Department of Psychology University of Waterloo Waterloo, ON Canada

**Keywords:** seniors, communication technology, social isolation, computers, intergenerational, older adults, mobile phone

## Abstract

**Background:**

Social integration and mental health are vital aspects of healthy aging. However, close to half of Canadians older than 80 years report feeling socially isolated. Research has shown that social isolation leads to increased mortality and morbidity, and various interventions have been studied to alleviate loneliness among older adults. This proposal presents an evaluation of an intervention that provides one-on-one coaching, is intergenerational, provides both educational and socialization experiences, and increases technology literacy of older adults to overcome loneliness.

**Objective:**

This paper describes the protocol of a randomized, mixed-methods study that will take place in Ontario, Canada. The purpose of this study is to evaluate if an intergenerational technology literacy program can reduce social isolation and depression in older adults via quantitative and qualitative outcome measures.

**Methods:**

This study is a randomized, mixed-methods, feasibility trial with 2 conditions. Older adults in the intervention condition will receive 1 hour of weekly technological assistance to send an email to a family member, for 8 weeks, with the assistance of a volunteer. Participants in the control condition will not receive any intervention. The primary outcomes are loneliness, measured using the University of California, Los Angeles Loneliness Scale, and depression, measured using the Center for Epidemiologic Studies Depression scale, both of which are measured weekly. Secondary outcomes are quality of life, as assessed using the Older People’s Quality of Life-Brief version, and technological literacy, evaluated using the Computer Proficiency Questionnaire-12, both of which will be administered before and after the intervention. Semistructured interviews will be completed before and after the intervention to assess participants’ social connectedness, familiarity with technology, and their experience with the intervention. The study will be completed in a long-term care facility in Southwestern Ontario, Canada. Significance was set at *P*<.05.

**Results:**

This study was funded in April 2019 and ethical approval was obtained in August 2019. Recruitment for the study started in November 2019. The intervention began in February 2020 but was halted due to the COVID-19 pandemic. The trial will be restarted when safe. As of March 2020, 8 participants were recruited.

**Conclusions:**

Information and communication technology interventions have shown varying results in reducing loneliness and improving mental health among older adults. Few studies have examined the role of one-on-one coaching for older adults in addition to technology education in such interventions. Data from this study may have the potential to provide evidence for other groups to disseminate similar interventions in their respective communities.

**International Registered Report Identifier (IRRID):**

DERR1-10.2196/23767

## Introduction

Social integration and mental health are fundamental indicators of healthy aging and represent significant public health concerns among Canadian older adults. Unfortunately, up to 50% of the Canadians older than 80 years report feeling socially isolated [1]. Similarly, 10%-15% of the Canadian older adults living in the community experience depression, and 44% of older adults in residential care have a diagnosis of depression or symptoms of depression [2,3]. Social isolation has significant detrimental effects on older adults, and previous research has shown an association between loneliness and depression [4-6]. Social isolation and higher levels of depressive symptoms, in turn, uniquely and jointly, are associated with increased morbidity and mortality, including reduced quality of life, decreased mental health, cognition, and function, and increased hospitalizations [1,7-12].

Multiple factors have been posited to influence loneliness, including health factors such as chronic diseases, in addition to cognitive and functional decline that may lead to difficulties in communication and mobility [1,13,14]. Structural factors that affect social isolation include relocation and separation from family and friends [15]. Given the multidimensional nature of loneliness, preventing and reducing loneliness is a top priority in Canadian public health care policy [1].

Growing evidence supports the use of intergenerational programs in improving various indicators for both older adults and younger cohorts. Interventions to reduce loneliness and improve mental health in older adults have traditionally consisted of group-based and individual-based interventions [16-19]. These include support groups, outreach volunteers, and animal therapy. In addition, previous studies have investigated information and communication technology methods such as internet-based videoconferencing and the overall impact of internet usage to maintain contact with family and friends, with varying levels of efficacy [20-22]. Previous mixed-methods intervention studies have identified improvements in social behavior, social interaction, quality of life, and mood in older adults [23]. These studies include production of art (eg, puppets or music), group reading, and shared spaces to gather with loved ones and friends [24-27].

Quantitative evidence also supports the use of intergenerational programs with older adults and youth, demonstrating efficacy via measures in geriatric depression scales, life satisfaction scales, quality of life, and self-esteem scales [28-34]. Examples of such studies include electronic gaming, student volunteer programs, and reminiscence therapy [29,35,36]. However, given the inherent social support that underlies intergenerational programs, it would be expected that improvements in the outcomes above are potentially mediated by reductions in social isolation and loneliness. Despite this, few studies have examined the role of intergenerational programming via a mixed-methods approach to reduce social loneliness [37-40]. As such, programs of this nature serve to improve not only relationships between older adults and younger adults but also the technological literacy and connectivity of older adults to family and friends via the internet—a multi-modal strategy to reduce social isolation and loneliness.

Addressing perceived social isolation or loneliness by using technology requires that older adults have a certain level of information and communication technology literacy, which is defined as the ability to locate, evaluate, and communicate information using a digital platform [18]. However, many older adults have low information and communication technology literacy, yielding a “digital divide” between older adults and younger adults in Canada, wherein older adults are less likely to use the internet as compared to younger adults [41]. Previous research has shown that compared to other age groups, older adults have less confidence in using technology and often need support for setup and use [42]. To target this, the enTECH Computer Club (enTECH) was founded in 2015 at the University of Waterloo. enTECH student volunteers provide one-on-one education to support older adults living in long-term care homes to use technology to keep in contact with their families, stay connected to their communities, and generally browse the internet. This student club consists of around 30 student volunteers from the undergraduate to graduate level who volunteer to work in service retirement homes and long-term care facilities in Southern Ontario. enTECH presents an opportunity for intergenerational learning.

This study is being conducted to determine the feasibility and effects of the educational program of enTECH for older adults on the levels of loneliness and depression reported by older adults. We hypothesize that our intervention will reduce loneliness and depression in older adults.

## Methods

### Managing Stakeholders

The organization implementing the intervention is a student club affiliated with the student union at the University of Waterloo, Ontario, Canada. We received funding through an internal funding opportunity. In order to proceed with this study, permission was required from both the student union and the institution’s research ethics board. The student union acknowledged that they lacked the resources and infrastructure to host the study and study funds, and thus, they requested that study funding and logistics be managed exclusively by a research team. This delineation has made managing the study easier by eliminating redundant paperwork, thereby allowing finances to be managed by research staff experienced in the required procedures and documentation.

### Study Design

This study is a mixed-method, randomized feasibility trial with 2 conditions. Participants in the intervention condition will receive assistance in sending an email to a designated family member once a week. This program will be carried out by experienced enTECH volunteers who have been trained in teaching older adults how to use technology. Participants in the control condition will continue interacting with their family as they do now (ie, not via information and communication technology). Participants in the control group also have access to 3 public computers in the facility and recreational therapists if they require support.

### Inclusion and Exclusion Criteria

As the focus of this study is specifically older adults who do not currently communicate with their family members in text by using technology, this is one of the key factors when determining the eligibility to participate. Potential participants are asked to confirm that they do not currently contact their family members using technology, including email, text message/iMessage, or any other digital text-based messaging platform (eg, WhatsApp, WeChat, Facebook Messenger). No exclusion criteria on the basis of computer proficiency will be applied, as the club provides support to people, regardless of their computer skills.

As this study is asking older adults to communicate with family members, participants were asked, prior to the consent process, if they had a family member who would agree to respond to an email from them weekly. No contact information will be collected at this time. There are no age or physical ability requirements to participate in this study, as long as the potential participant resides in a long-term care home. There are no exclusion criteria based on previous information and communication technology experience or expertise. If a participant is unable to physically use the technology to send an email, a member of the research team will assist them with doing so. Staff at the location will be asked to both identify potential participants and residents of that location who may not be a good fit to participate. Participants will be excluded if they have cognitive impairment with the inability to consent. Participants who are unable to speak English will be excluded from this study.

### Ethics Approval

This study was approved by the University of Waterloo Office of Research Ethics as of September 2019. The study ID is ORE #41104.

### Randomization and Blinding

After the consenting process, participants will be randomized to either the intervention or the control group by using a computer-generated allocation sequence in a 1:1 ratio. If randomized to the intervention group, researchers will work with the participants to obtain a family member’s email address for use in the study. Owing to the nature of the study, participants cannot be blinded to allocation. Two separate research teams will facilitate the study. The clinical team will be carrying out the intervention, while the research team will be conducting the interviews and administering the scales. In order to maintain participant confidentiality and anonymity to the interviewers, participants will be assigned a participant ID number via the allocation sequence described above. This number will be known to both the clinical team and the research team; however, the research team will not be able to see the participants in the intervention group or those in the control group. When data are entered into the computer, the data will be associated with the participant IDs alone.

### Recruitment

#### Location Recruitment

Recreation facilitators at the nursing home will be contacted and asked for permission to run the study at their location via the location recruitment letter (Multimedia Appendix 1). Recreation facilitators will be contacted by email using a secure email specific for study purposes, in addition to informal communication methods (eg, in-person conversations) between the recreation facilitator and the study team.

#### Participant Recruitment

Participants will be recruited through a long-term care home in Southern Ontario. Recreational facilitators will assist in recruiting participants for the study. Moreover, brochures, pamphlets, and flyers (Multimedia Appendix 2) will be placed in the study location by the team, in addition to a town hall advertising the study. Potential participants will be asked 2 questions to determine eligibility: if they currently use email/texting/messaging to contact family members and if they believe a family member would be willing to respond to an email sent by them once a week (Multimedia Appendix 3). Collecting the contact information of the participants’ family members requires balancing of respect for the participants’ autonomy, along with their potential comfort with the study’s subject matter. After consenting, participants will be presented with 2 options so that the research team can contact their family members. One option will consist of the participant providing the research team with a ranked list of family members to potentially receive emails from the participant, along with their phone numbers. The other option will consist of providing the participants with an information sheet, written in layperson terms, which they can use to describe the study to the family members to ask if they would like to participate. Members of the research team will follow up after 3 days to see if the participants were able to contact their family members or to provide support to facilitate this contact. The research team will then offer to contact the family member on their behalf, and at such time will record the contact information of up to 3 family members and contact them using the telephone script (Multimedia Appendix 4). Recruitment for the study started in November 2019. We expected to enroll approximately 20 participants, 10 in each condition. We successfully enrolled 8 participants before the study was forced to halt due to the COVID-19 pandemic (Multimedia Appendix 5).

#### Student Recruitment

The study team will consist of 10 students from the University of Waterloo who are volunteers in the enTECH Computer Club. Student volunteers were recruited through school club advertisements, course announcements, and through social media. Student volunteers were interviewed and trained to provide technology education through in-house lesson plans and simulations.

### enTECH Intervention and Study Procedure

**Figure 1:**
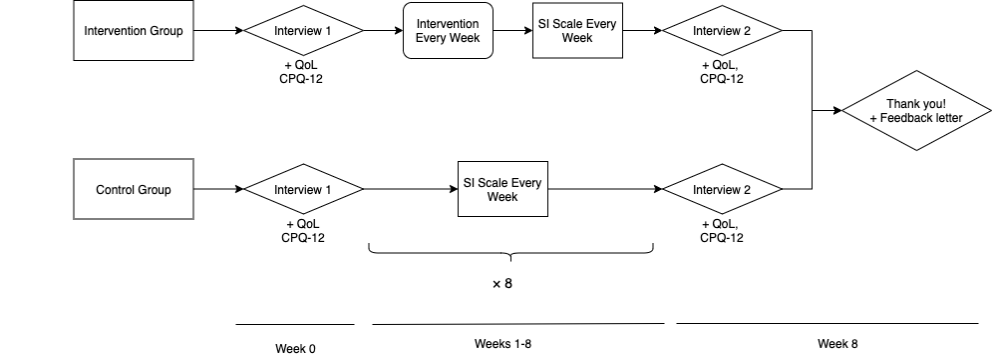
Diagram detailing the timeline for the study components. QoL: quality of life; CPQ-12: Computer Proficiency Questionnaire-12; SI scale: social isolation scales (Center for Epidemiologic Studies Depression Scale and University of California, Los Angeles Loneliness Scale version 3).

After obtaining informed consent, demographic information will be collected (Multimedia Appendix 6) and participants will be randomized as described above. The intervention outline is described below and detailed in the study flow chart of Figure 1.

*Week 0*: Members of the study team will conduct semistructured interviews with participants (Multimedia Appendix 7) and complete the Computer Proficiency Questionnaire-12 (CPQ-12) and the Older People’s Quality of Life-Brief version (OPQOL-Brief) questionnaire, which will be measured before and after the study period.

*Weeks 1-7:* Participants in the intervention group will complete the enTECH program. Trained enTECH volunteers will help older adults use either our laptops or participants’ own technology systems to email their family members. These sessions will take place in group settings in a public area in the long-term care facility accessible to all participants. Both the Center for Epidemiologic Studies Depression scale (CES-D) and the UCLA (University of California, Los Angeles) Loneliness Scale (version 3) will be measured each week in the intervention and control groups.

*Week 8:* Participants in both groups will complete their final session of the intervention, and they will then be asked to complete the CPQ-12, the OPQOL-Brief, the CES-D, and the UCLA loneliness scale. Participants in the intervention group will also be invited to complete a second interview discussing their experiences using technology to communicate with a family member (Multimedia Appendix 8).

### Outcomes

*Primary outcomes:* The primary outcomes of this study are loneliness and depression. Loneliness will be assessed using the UCLA Loneliness Scale-version 3 [43]. The scale is a 20-item measure using a 4-point Likert scale and has been validated for use with older adults [44]. Depression will be measured using the CES-D [45]; this is a 20-item scale, each with 4 response items ranked from 0 to 3 based on the frequency of symptoms associated with depression over the past week. A cut-off of over and equal to 16 has been used in multiple validation studies [46].

*Secondary outcomes*: The secondary outcomes of this study are quality of life and computer proficiency. Quality of life will be measured using the OPQOL-Brief and a 13-item Likert scale from 1 to 5. This scale has been validated in older adults [47]. Technological proficiency will be measured using the CPQ-12, a 12-item measure using a 1-5 Likert scale and has been well-studied in older adults [48].

*Feasibility outcomes:* The feasibility outcome measures of this study will include the number of participants that we are able to enroll in the study, adherence to the intervention (frequency of contact with the family and attendance to the program), and the rate of attrition [49]. We also intend to determine the challenges faced by the site and volunteers as a result of our intervention, in particular, the restrictions of having only university student volunteers as well as the restrictions of the daily schedules for the residents of the facility. These will both be determined quantitatively (eg, total number of participants, adherence) and qualitatively, where participants will be asked about their experiences with the program. We do not expect any adverse events as a result of our intervention.

*Qualitative interviews:* Semistructured interviews will be conducted with older adults prior to and following the 8-week intervention. Interviews will be continued with all the participants before the study and those in the intervention condition after the study has concluded. Interviews will be digitally audio recorded and transcribed verbatim, and pseudonyms will be assigned to participants. A thematic analysis methodology will be used to code the qualitative data. Two independent investigators will conduct line-by-line coding. The preintervention and postintervention questionnaires are provided in Multimedia Appendix 7 and Multimedia Appendix 8, respectively. Qualitative interviews will provide context in the role that the enTECH program provides for older adults living in long-term care facilities as well as how they currently socialize with others. By assessing participants both before and after the intervention, we hope to provide narrative data on how the enTECH program may have changed the way participants socialize in their community, determined changes in their knowledge with technology, and provided insight into their experience with the program. In addition, by understanding the goals participants have by enrolling in the program, future modifications to the program may allow for more tailored technology and social interventions.

### Statistical Analysis

#### Statistical Software

Participant demographic data will be tabulated for both intervention and control groups with their respective means and standard deviations. Participant dropouts will also be considered, and an intent-to-treat analysis will be completed. All data will be analyzed using GraphPad Prism 9 (GraphPad Software, Inc).

#### Primary Outcomes

The UCLA Loneliness Scale and the CES-D Scale will be analyzed using a repeated measures approach, as these measures will be collected weekly. The normality of the data will be assessed using the Shapiro-Wilk test; if the data are nonparametric, a Q-Q plot will be performed to identify skewness of the data. A Brown-Forsythe test will be used to determine equal variance. If the data are determined to be normally distributed and equal variance is observed between the groups, a repeated measures analysis of variance test will be used to detect between-group and within-group differences. If normality cannot be established or unequal variance is observed within the data, the Friedman test will be used to detect between-group and within-group differences. If the data are nonnormal with unequal variance, a Box-Cox transformation will be performed to obtain equal variances. For all tests, statistical significance will be set at *P*<.05.

#### Secondary Outcomes

The OPQOL-Brief and the CPQ-12 will be analyzed by comparing changes in the scores between the intervention group and the control group. The normality of the data will be assessed using the Shapiro-Wilk test, and equality of the variances between the groups will be assessed via a Q-Q plot and the Brown-Forsythe test. The differences between prestudy and poststudy period assessments in the intervention and control groups will be assessed using pooled variances and a one-sided *t* test. If normality cannot be established or unequal variance is observed within the data, a Q-Q plot will be used to determine the appropriate test (eg, Sign test, Wilcoxon-Signed Rank Test, or Wilcoxon Rank-Sum Test). For all tests, statistical significance will be set at *P*<.05. Quantitative feasibility outcomes (eg, adherence and attrition) will be reported as descriptive data and compared using a one-sided *t* test or a nonparametric equivalent (Mann-Whitney *U* test). All evaluation outcomes will be integrated in a joint display.

#### Power

This study was designed as a feasibility study for investigating the feasibility of a student-run initiative to reduce loneliness in older adults. A component of this study will be understanding recruitment and retention, which may have significant variability in our sample size. Based on previous studies on interventions to reduce loneliness in older adults, with an effect size of approximately 0.2 and alpha of .05 and 80% power, we expect our sample size to be 196 [43,50-54]. Accounting for attrition rates in other intergenerational programs would be approximately 10%, and the sample size would be 216 [20,55,56]. Since the nature of this study is a feasibility study, which we aim to use to guide a future randomized controlled trial, this feasibility trial is not registered.

## Results

### Participant Recruitment

**Figure 2:**
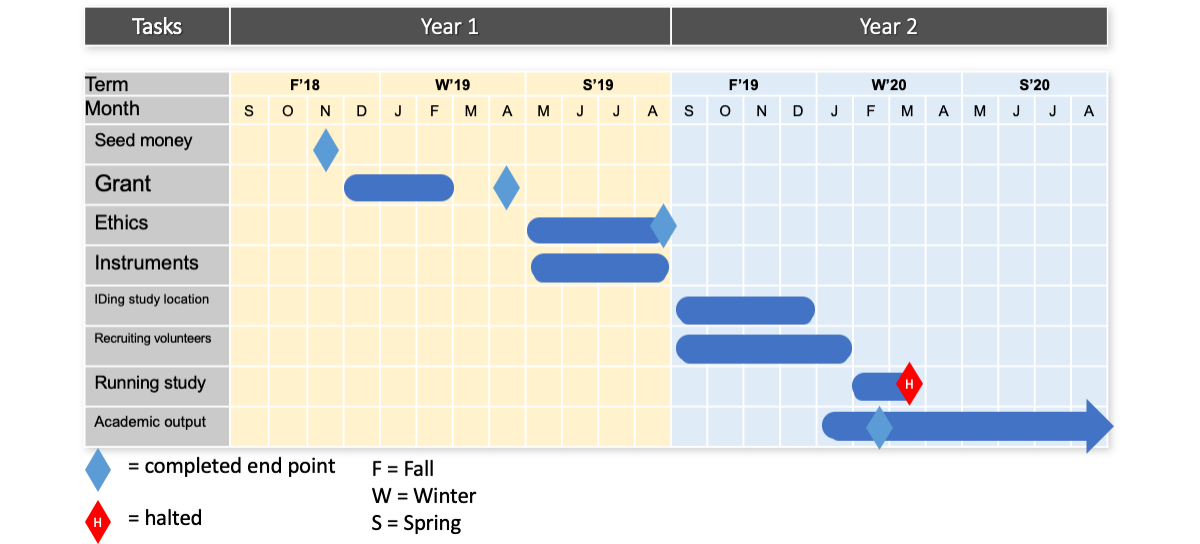
Gantt chart for currently completed pilot.

**Figure 3:**
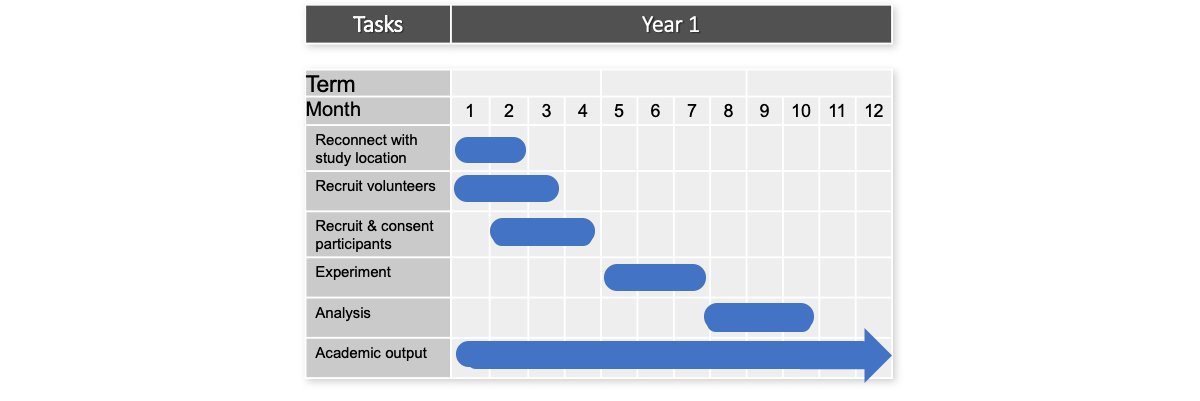
Gantt chart for when the pilot will be restarted.

Recruitment started in November 2019. As of March 2020, we have recruited 8 participants. This study was halted as per the University of Waterloo Office of Research Ethics in accordance with public health guidelines. We expect that recruitment will be resumed once the long-term care facility can offer the program for older adults, in conjunction with approval from the ethics board to ensure safety for both students and participants. Gantt charts are provided detailing our progress to date Figure 2, which details our progress to date (Figure 2) and the expected timeline once permitted to resume (Figure 3).

### Evaluation Outcomes

Demographic outcomes will be tabulated, as shown in Table S1 of Multimedia Appendix 9. A joint display will be used to integrate qualitative and quantitative data [57,58]. This joint display will present test statistics for the 4 variables measured as well as exemplary quotes from the intervention group before and after the study (Table S2 of Multimedia Appendix 9). There will also be a column to highlight the results of integrating the qualitative and quantitative data. Feasibility outcomes will be displayed, as shown in Table S3 of Multimedia Appendix 9.

## Discussion

### Overview of the Study

We were able to recruit 8 participants, before the study was forced to halt due to the COVID-19 pandemic, in accordance with institutional and public health guidelines. A large component of this study involves coordinating the schedules of student volunteers, older adults, and staff at the long-term care home. This presents a number of challenges as in-person classes lead to students generally being available only in the evenings, older adults often have busy schedules, and staffing shortages are prevalent in Canadian long-term care locations [59]. Scheduling is anticipated to be a problem, which will be resolved through extensive dialogue and trial-and-error scheduling. 

The enTECH Computer Club in this study is a club with the Waterloo Undergraduate Student Association (WUSA), which does not have the infrastructure to manage the awarded research funding. Additionally, club activities require approval from the WUSA. In consultation with WUSA, the decision was made to run this study independent of WUSA, so as to use existing university research infrastructure. The club was referenced to establish a basis for this study to indicate the program used and the expected backgrounds of the research assistants, but this does not indicate that the club is otherwise affiliated with this study.

### Principal Results

This study will contribute to a deeper understanding of intergenerational technology programs for older adults living in long-term care homes. We hope that these data will allow for stronger advocacy efforts for the benefits these programs can provide for older adults, especially in improving their mental health and reducing their feelings of loneliness. We also hope that this protocol will allow others who wish to study university club programs as a starting point on how to do so. Navigating the requirements for university clubs, which are generally affiliated with university student unions, as well as university research requirements, including institutional review board approval, is a first-time occurrence for our institution from our understanding.

### Limitations

Obtaining a sufficiently large sample to observe statistical significance for the measures used is anticipated to be a challenge, as resource limitations necessitate that this study is only run at 1 location. Should additional funding be secured, there is a potential for this study to be run at multiple sites.

### Conclusions

We hope that the results of this study will highlight the need for increases in innovative technologically focused programs for older adults and the need for additional resources to be directed to promoting the value of this program to older adults.

## Appendix

Multimedia Appendix 1 Location recruitment letter.

Multimedia Appendix 2 Advertisement to older adults.

Multimedia Appendix 3 Screening questions.

Multimedia Appendix 4 Email script for phone call with family member.

Multimedia Appendix 5 CONSORT 2010 flow diagram for the study.

Multimedia Appendix 6 Demographics questionnaire.

Multimedia Appendix 7 Preprogram interview guide.

Multimedia Appendix 8 Postprogram interview guide.

Multimedia Appendix 9 Proposed tables for recording evaluation outcomes.

## Abbreviations

CES-D: Center for Epidemiologic Studies Depression scale
CPQ-12: Computer Proficiency Questionnaire-12
OPQOL-Brief: Older People’s Quality of Life-Brief version
UCLA: University of California, Los Angeles
WUSA: Waterloo Undergraduate Student Association
