# Supplementary material for: Evaluation of an Intergenerational and Technological Intervention for Loneliness: Protocol for a Feasibility Randomized Controlled Trial
Source: JMIR Res Protoc. 2021 Feb 17;10(2):e23767. doi: 10.2196/23767 (PMC7929741; doi:10.2196/23767)
Supplement: Multimedia Appendix 1 [file resprot_v10i2e23767_app1.pdf]

UNIVERSITY OF
WATERLOO

DATE

Dear NAME:

This letter is a request for [location]'s assistance with a project I am conducting under the supervision of Dr. Kelly Grindrod, Dr. Karla Boluk, and Dr. Uzma Rehman at the University of Waterloo.

The purpose of this study is to investigate the use of technology to connect older adults to their families, to help them feel less isolated from their family. We also want to determine how having younger people helping older people stay connected to their families impacts the older people. We will be studying this by teaching older adults how to use a computer (either their own, or that we bring) to send emails to a family member. We will also collect information on mood, quality of life, and ability to use a computer. Not all older adults who indicate they are interested in participating will receive instruction: we aim to have a control group who we ask questions about their mood weekly, but who do not participate in the programming.

This study will ask older adults to provide the name of family members who may be interested in emailing the older adult. After receiving consent to do so from the family member, research assistants wish to hold sessions once a week at [location] where the older adults will be supported in using email to contact their family member. At the conclusion of these sessions, the older adults will be asked questions about their mood and experiences of social isolation. We aim to run the sessions for 8 weeks total.

To respect the privacy and rights of [location], I will not be contacting the residents directly. What I intend to do is provide [location] with information flyers to be distributed by you at your discretion. Members of the research team would also be happy to go to your location and conduct a "town hall" style meeting, where we discuss the study with your residents. Contact information for my advisors and me will be contained on the flyers or packages. If a resident is interested in participating, they will be invited to contact us who will generate and submit a list of potential participants to [location]. In collaboration with you, it will be ensured that all participants have the capacity to consent to participation in such a study. If [location] has a preferred study recruitment procedure please advise and I will be happy to follow the standard procedure.

This study will participants to think about if they are lonely or feeling depressed. All of the researchers who interact with the participants will have been trained on appropriate interviewing skills, and all of the questionnaires that we will use have been designed for use with older adults. If {location} is interested in assisting with this study, we would ask for information on how participants are able to access psychological support at {location}. Additionally, participants may contact their family physician, or the Here 24/7 helpline at 1-844-437-3247. Participants are free to skip any questions and can withdraw from the study at any time.

UNIVERSITY OF WATERLOO
CONCORDIA CUM VERITATE

This study has been reviewed and received ethics clearance through a University of Waterloo Research Ethics Committee (ORE# 41104). If you have questions for the Committee contact the Office of Research Ethics, at 1-519-888-4567 ext. 36005 or ore-ceo@uwaterloo.ca.

If you have any questions regarding this study or would like additional information to assist you in reaching a decision about participation, please contact me at (519) 804-6984 or by email at computing.study@uwaterloo.ca. You may also contact any of the faculty investigators listed at the bottom of this letter.

I hope that the results of my study will be beneficial to [location], to other long term care and community facilities, as well as the broader research community.
I very much look forward to speaking with you and thank you in advance for your assistance with this project.

Yours sincerely,

Colin R.J. Whaley, MSc(c), BSc
Masters Student
School of Pharmacy
University of Waterloo

Venus Ho, BA(c)
Undergraduate Student
Department of Psychology
University of Waterloo

Kelly Grindrod, PharmD, MSc
Associate Professor
School of Pharmacy
University of Waterloo

519-888-4567 ext. 21358,
kelly.grindrod@uwaterloo.ca

Uzma Rehman, PhD, CPsych
Clinical Psychologist
Associate Professor
Department Psychology
University of Waterloo

519-888-4567 ext. 38361,
rehman@uwaterloo.ca

Karla Boluk, PhD
Assistant Professor
Department of Recreation and Leisure Studies
University of Waterloo

519-888-4567 ext. 34045,
kboluk@uwaterloo.ca

Agreement Form

We agree to help the researchers recruit participants for this study from [location].

□ YES □ NO

We agree to the use of the name of [location] in any thesis or publication that comes of this research.

□ YES □ NO

If NO, a pseudonym will be used to protect the identity of the organization.

Director Name: ________________________________ (Please print)

Director Signature: _____________________________

Board of Directors Representative Name: ________________________________ (Please print)

Board of Directors Representative Signature: ____________________________

Witness Name: __________________________________ (Please print)

Witness Signature: ______________________________

Date: ____________________________
