# Supplementary material for: Evaluation of an Intergenerational and Technological Intervention for Loneliness: Protocol for a Feasibility Randomized Controlled Trial
Source: JMIR Res Protoc. 2021 Feb 17;10(2):e23767. doi: 10.2196/23767 (PMC7929741; doi:10.2196/23767)
Supplement: Multimedia Appendix 2 [file resprot_v10i2e23767_app2.pdf]

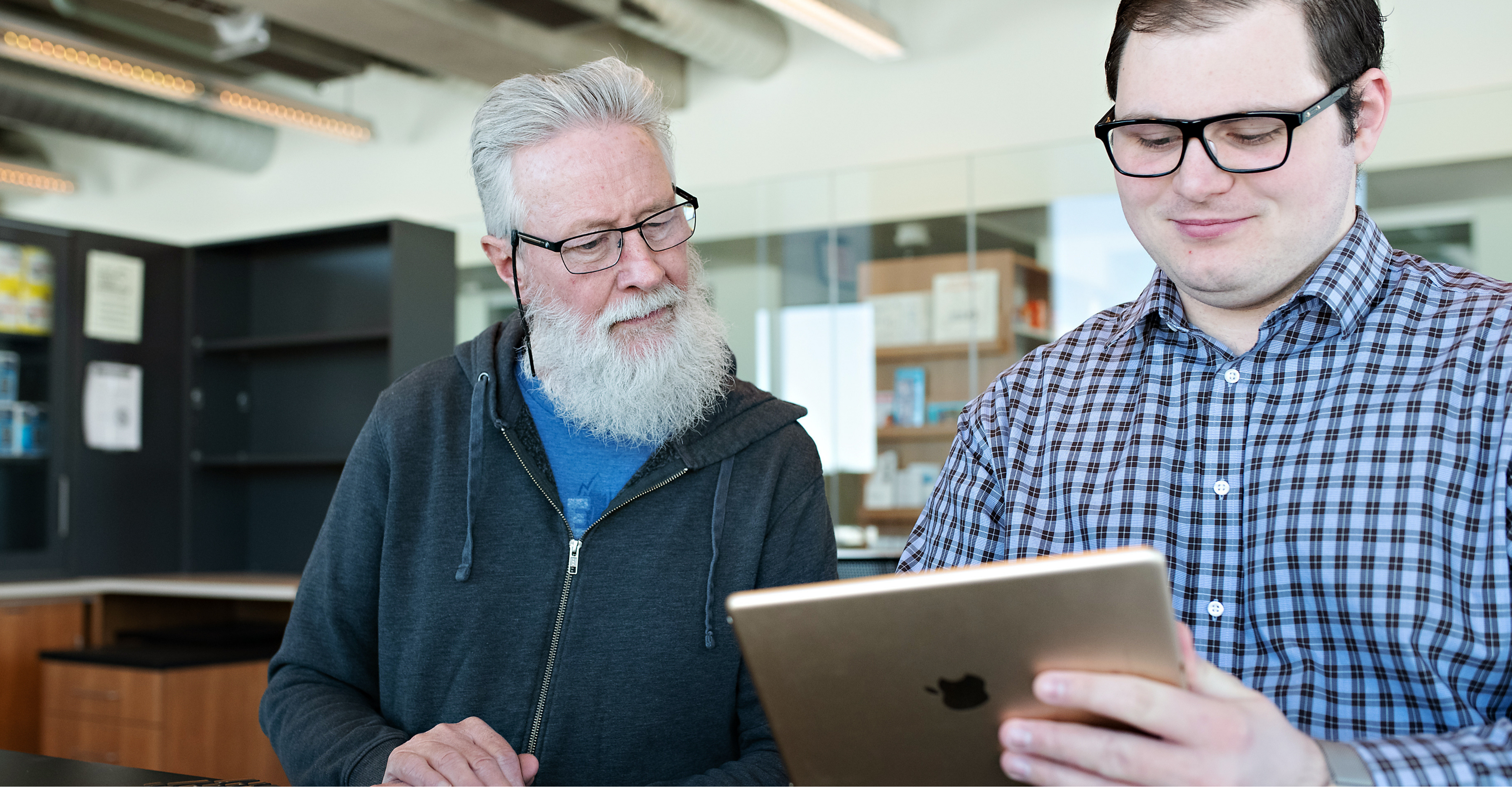

UNIVERSITY OF WATERLOO

PARTICIPANTS NEEDED FOR RESEARCH IN USING COMPUTERS TO CONNECT TO FAMILY MEMBERS

Learn how to connect with your family members over email!

CALL or EMAIL
(519) [redacted]
[redacted]@uwaterloo.ca

This study has been reviewed by, and received ethics clearance through a University of Waterloo Research Ethics Committee.
ORE #41104

- Sessions once a week, around 1 hour per week
- Device loan and training provided for free
- We will ask you some questions about your experience with technology, and your mood
- NO computer experience needed
- **Must not be currently emailing family members**
