# Supplementary material for: Evaluation of an Intergenerational and Technological Intervention for Loneliness: Protocol for a Feasibility Randomized Controlled Trial
Source: JMIR Res Protoc. 2021 Feb 17;10(2):e23767. doi: 10.2196/23767 (PMC7929741; doi:10.2196/23767)
Supplement: Multimedia Appendix 3 [file resprot_v10i2e23767_app3.pdf]

UNIVERSITY OF WATERLOO

Screening questions:

1. In order to best determine your eligibility to participate in this study, we have some questions to ask you.

Do you currently use technology in any way to keep in contact with your family **regularly**? In this case, regularly means at least once a week over the last month. This includes email, text messages/texting, or any sort of messaging application (e.g. WhatsApp, Facebook Messenger, WeChat)?

Please circle your answer:

YES NO

2. In order to be a part of this study, a family member will need to respond to emails you send them, which volunteers will help you do. **Do you believe that a family member of yours would be willing to respond to an email sent by you once a week?** The research team will need to contact them to gain their approval to be a part of the study.

Please circle your answer:

YES NO
