# Supplementary material for: Evaluation of an Intergenerational and Technological Intervention for Loneliness: Protocol for a Feasibility Randomized Controlled Trial
Source: JMIR Res Protoc. 2021 Feb 17;10(2):e23767. doi: 10.2196/23767 (PMC7929741; doi:10.2196/23767)
Supplement: Multimedia Appendix 4 [file resprot_v10i2e23767_app4.pdf]

Email script for phone call with family member

P = Potential Participant; I = Interviewer

I - May I please speak to [name of potential participant]?

P - Hello, [name of potential participant] speaking. How may I help you?

I - My name is [insert researcher name] and I am a research assistant at the University of Waterloo. Your [relation] [name] has indicated that you may be interested in participating in a study with [him/her/them]. I am currently conducting research under the supervision of Professors Kelly Grindrod, Karla Boluk and Uzma Rehman on the experiences of older adults living away from their families. The study involves connecting older adults who live away from their families to a family member via email. As a part of this research, we have contacted your [family member: specify parent/aunt/uncle] at [location], and they indicated that you may be interested in emailing your [relation] as a part of this study.

Is this a convenient time to give you further information about the study? This call should take five minutes.

P - No, could you call back later (agree on a more convenient time to call person back).

OR

P - Yes, could you provide me with some more information regarding the interviews you will be conducting?

I - Background Information:

- The study involves having university students help connect older adults living away from their family members using email. We are studying if this type of programming is effective in helping reduce the older adults' feelings of loneliness.
- We will be going to your family member's [retirement/long-term care] home and helping teach them how to use computers to support them in contact a family member using email. This study will go on for 8 weeks; we will support them during that time.
- We are asking if you would be willing to provide your email address to us, so we can support your family member in emailing you, and we would ask that you respond to your [relation i.e. mother/father/etc.]'s email within one week.
- None of the contents of the emails will be used for the study in any way, and the extent of your involvement would be responding to these emails.
- If you provide your email address, we will:
  - send you a summary of our conversation today
  - email you when the study is beginning with your [family member]'s email address
  - let you know when the study is concluding
  - will never share your information outside of the research team under any circumstance

- We anticipate beginning [date].
- If you have any questions regarding this study or would like additional information to assist you in reaching a decision about participation, please feel free to contact Dr. Kelly Grindrod at 519-888-4567, Ext. 21358.
- I would like to assure you that this study has been reviewed and received ethics clearance through a University of Waterloo Research Ethics Committee. However, the final decision about participation is yours.
- Do you consent to receiving emails from us and your [relation] for this study?

P - No thank you.

OR

P - Yes

I - Thank you. What email address would you like your us and your [relation] to contact you at?

P – name@example.com

I - Thank you. We will email you right after this conversation with a summary of our discussion. We anticipate you should receive an email from your family member by [date]. Please know you can contact us by phone or email at any time.

P - Good-bye.

I - Good-bye.
