# Supplementary material for: Evaluation of an Intergenerational and Technological Intervention for Loneliness: Protocol for a Feasibility Randomized Controlled Trial
Source: JMIR Res Protoc. 2021 Feb 17;10(2):e23767. doi: 10.2196/23767 (PMC7929741; doi:10.2196/23767)
Supplement: Multimedia Appendix 5 [file resprot_v10i2e23767_app5.doc]

**
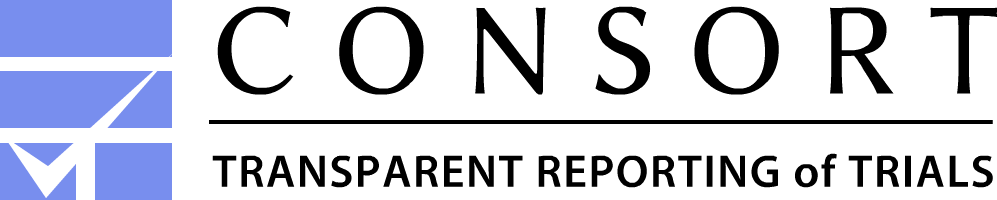
**

**CONSORT 2010 Flow Diagram**

**Allocation**

**Analysis**

**Follow-Up**

**Enrollment**

Assessed for eligibility (n= 9)

Excluded (n= 1)

♦  Declined to participate (n= 1)

Analysed (n= 0)
♦ Excluded from analysis (n= 0)

Lost to follow-up (give reasons) (n= 0)

Discontinued intervention (n= 0)

Allocated to intervention (n= 4)

♦ Received allocated intervention (n= 0 )

♦ Did not receive allocated intervention (COVID-19*) (n= 4)

Lost to follow-up (n= 0)

Discontinued intervention (n= 0)

Allocated to intervention (n= 4)

♦ Received allocated intervention (n= 0)

♦ Did not receive allocated intervention (COVID-19*) (n= 4)

Analysed (n= 0)
♦ Excluded from analysis (n= 0)

Randomized (n= 8)

*note: the study stopped in March 2020 due to the COVID-19 pandemic
