# Supplementary material for: Evaluation of an Intergenerational and Technological Intervention for Loneliness: Protocol for a Feasibility Randomized Controlled Trial
Source: JMIR Res Protoc. 2021 Feb 17;10(2):e23767. doi: 10.2196/23767 (PMC7929741; doi:10.2196/23767)
Supplement: Multimedia Appendix 6 [file resprot_v10i2e23767_app6.pdf]

Number: ___ ___ ___-___ ___ ___
Date: ___ ___/___ ___/ ___ ___

Demographics Questionnaire

Part 1: When asking about conditions below, frame in patient-friendly language e.g., have you ever been diagnosed with depression? If the participant is unsure, check no.

1. Age (years): ___________

2. Gender: _______________

3. How often do you **talk to** family members that do not live with you on the phone/via video chat? (E.g. children, grandchildren, nieces/nephews.)
   - ☐ Once per day
   - ☐ More than once per week
   - ☐ Once per week
   - ☐ Two-three times per month
   - ☐ Once a month
   - ☐ Less than once per month

4. How often do you **see** family members that do not live with you in person? (E.g. children, grandchildren, nieces/nephews.)
   - ☐ Once per day
   - ☐ More than once per week
   - ☐ Once per week
   - ☐ Two-three times per month
   - ☐ Once a month
   - ☐ Less than once per month

5. When was the last time you **lived with** other family members? (E.g. your siblings, children, grandchildren, nieces/nephews.)
   - ☐ Less than 6 months
   - ☐ 6 months to 1 year
   - ☐ 1 year to 3 years
   - ☐ 3 years to 5 years
   - ☐ More than 5 years ago

6. Has a doctor ever told you that you have depression?
   - ☐ Yes
   - ☐ No

7. Has a doctor ever told you that you have an anxiety disorder?
   - ☐ Yes
   - ☐ No

Number: ____ ____ ____-____ ____ ____
Date: ____ ____/____ ____/ ____ ____

Part 2: The following section asks for basic information about you including your past experience with computers.

1. What best describes your employment status?
   - ☐ Employed/Working full-time
   - ☐ Employed/Working part time
   - ☐ Retired
   - ☐ Never worked/managed a household
   - ☐ Other

2. If you answered "Retired" for Question 1, how long have you been retired for? Please write the number in the space below:

   _______ years

3. While employed, how often did you use a computer?
   - ☐ Daily
   - ☐ Weekly
   - ☐ Monthly
   - ☐ Rarely
   - ☐ Never

4. How often do you currently use a computer?
   - ☐ Daily
   - ☐ Weekly
   - ☐ Monthly
   - ☐ Rarely
   - ☐ Never

5. How skilled are you with using a computer?
   - ☐ Non-user - Not skilled at all
   - ☐ Novice - Slightly skilled
   - ☐ Intermediate - Somewhat skill
   - ☐ Expert - Very skilled

6. Do you use a computer at home or at work?
   - ☐ Yes
   - ☐ No (skip to question 7)

7. How often do you use your computer?
   - ☐ Daily
   - ☐ Weekly
   - ☐ Monthly
   - ☐ Rarely

Number: ____ ____ ____-____ ____ ____
Date: ____ ____/____ ____/ ____ ____

8. Do you own a tablet like an iPad?
   - ☐ Yes
   - ☐ No (skip to question 9)

9. How often do you use your handheld computer?
   - ☐ Daily
   - ☐ Weekly
   - ☐ Monthly
   - ☐ Rarely

10. Do you own a smartphone with a touch screen like an iPhone or an Android (Samsung)?
    - ☐ Yes
    - ☐ No (skip to question 11)

11. How often do you use your smartphone?
    - ☐ Daily
    - ☐ Weekly
    - ☐ Monthly
    - ☐ Rarely

12. What is your highest level of education?
    - ☐ High school
    - ☐ Trade school
    - ☐ College
    - ☐ University
    - ☐ Graduate Degree (MSc, MA, PhD)
    - ☐ Professional Degree (MD, BScPharm, MBA)

13. We would like to ask you about your personal income because it affects whether you can afford things like computers. You can choose not to answer. Here are a few income ranges. Which one best describes your yearly household income before taxes and deductions, from all income sources?

    - ☐ Less than $10,000
    - ☐ $10,000 to $19,999
    - ☐ $20,000 to $29,999
    - ☐ $30,000 to $39,999
    - ☐ $40,000 to $49,999
    - ☐ $50,000 to $59,999
    - ☐ $60,000 to $69,999
    - ☐ $70,000 to $79,999
    - ☐ $80,000 to $89,999
    - ☐ $90,000 to $99,999
    - ☐ $100,000 to $149,999
    - ☐ $150,000 or more
    - ☐ Choose not to answer

This is the end of the questionnaire, thank you for participating.
