# Supplementary material for: Evaluation of an Intergenerational and Technological Intervention for Loneliness: Protocol for a Feasibility Randomized Controlled Trial
Source: JMIR Res Protoc. 2021 Feb 17;10(2):e23767. doi: 10.2196/23767 (PMC7929741; doi:10.2196/23767)
Supplement: Multimedia Appendix 7 [file resprot_v10i2e23767_app7.pdf]

Appendix: Interview Guides
Pre-Program Interview Guide: Participants

2. How often do you interact with others?
    a. Are there times when you have visitors? Are these typically scheduled or do friends and family drop in and visit you?
    b. Social isolation is defined as a lack of social and emotional attachments to others and is characterized by feelings of loneliness (Moyle, Kellett, Ballantyne, & Gracia, 2011). Have you ever experienced feelings of social isolation and/or depression? If so, can you speak to this a bit more?
    c. (If answered yes to previous question) How have you responded to feelings of social isolation and depression in the past?
3. Do you feel connected to your retirement community? If yes, how so?
    a. Are there times when you leave the retirement community for socialization? If so, can you provide me with an example?
    b. Are there any barriers that stop you from participating in your community?
4. What are some hobbies and/or activities you engage in, in your community?
    a. Where do you carry out these activities/hobbies?
    b. How often do you do these activities?
    c. Who do you do these activities with?
    d. How did you find out about these activities?
5. How familiar are you with technology?
    a. How long have you been using technology?
        i. Were you exposed to technology in your career? If so, can you provide me with an example?
    b. What kinds of technology do you use? (e.g. computer, laptop, cellphone, iPad)
    c. Do you have access to pieces of technology? If so, where do you access this technology? (personal, library, LTC, donation, etc.)
    d. What sorts of things do you do with technology? (e.g. emails, Facebook, Skype)
    e. Does technology allow you to connect with others?
    f. Are there any challenges you face when using technology? If so, what are they?
6. You have enrolled with the enTECH program, what drew you to this program?
    a. What are you hoping to get out of this program?
    b. Have you experienced a program like this before? If so, how was it similar or different?
7. Is there anything else that you would like to share?
