# Supplementary material for: Evaluation of an Intergenerational and Technological Intervention for Loneliness: Protocol for a Feasibility Randomized Controlled Trial
Source: JMIR Res Protoc. 2021 Feb 17;10(2):e23767. doi: 10.2196/23767 (PMC7929741; doi:10.2196/23767)
Supplement: Multimedia Appendix 8 [file resprot_v10i2e23767_app8.pdf]

Post- Program Interview Guide: Participants

1. Tell me about your experience with the enTECH program?
    a. Can you provide me with an example of what you did in an enTECH session?
        i. What was that experience like for you?
    b. What did you like most about the program?
    c. Is there anything you would recommend changing about the program in order to improve it?
    d. Did you experience any challenges in participating in this program? If so, what were they?
    e. Were you supported in this program? If so, in what ways were you supported?
    f. Can you describe if there was anything you specifically looked forward to in each of the enTECH sessions?
    g. Has your involvement in the program improved the way you feel in any way?
2. Has participating in this program changed how you feel regarding your connection to the community?
    a. Were there any barriers you experienced that limited you to feel connected?
    b. Were there any supports that you experienced that supported your connection to the community?
    c. Has your connection with members of your community changed now after the program. If so, how?
    d. Have you established any connections as a result of participating in this program?
        i. With other residents?
        ii. With young volunteers?
        iii. With family members?
3. What have you learned from participating in this program?
    a. What sorts of things can you do with technology?
    b. How confident do you feel in using technology now? Why?
    c. Do you have access to technology to carry on your learnings now that the program has finished?
4. Is there anything else you would like to share about your experiences in the enTECH program?
