# Supplementary material for: Evaluation of an Intergenerational and Technological Intervention for Loneliness: Protocol for a Feasibility Randomized Controlled Trial
Source: JMIR Res Protoc. 2021 Feb 17;10(2):e23767. doi: 10.2196/23767 (PMC7929741; doi:10.2196/23767)
Supplement: Multimedia Appendix 9 [file resprot_v10i2e23767_app9.docx]

**Tables that will be used in the study process.**

**Table S1.** Proposed demographic outcomes.

|  | Intervention (mean, SD) | Control (mean, SD) |
| --- | --- | --- |
| Age |  |  |
| Gender (percentage, absolute number) |  |  |
| How often do you talk to family members that do not live with you on the phone/via video chat? (percentage, absolute number) |  |  |
| How often do you see family members that do not live with you in person? (percentage, absolute number) |  |  |
| When was the last time you lived with other family members? (percentage, absolute number) |  |  |
| Diagnosed depression (percentage, absolute number) |  |  |
| Diagnosed anxiety disorder (percentage, absolute number) |  |  |
| Employment status (percentage, absolute number) |  |  |
| Number of years retired |  |  |
| While employed, how often did you use a computer? (mean, SD) |  |  |
| How often do you currently use a computer? (percentage, absolute number) |  |  |
| How skilled are you with a computer? (percentage, absolute number) |  |  |
| Do you use a computer at home or at work? (percentage, absolute number) |  |  |
| How often do you use your computer? (percentage, absolute number) |  |  |
| Do you own a tablet? (e.g. iPad) (percentage, absolute number) |  |  |
| How often do you use your handheld computer? (percentage, absolute number) |  |  |
| Do you own a smartphone with a touchscreen? (e.g. iPhone or Android device) (percentage, absolute number) |  |  |
| How often do you use your smartphone? (percentage, absolute number) |  |  |
| What is your highest level of education? (percentage, absolute number) |  |  |
| What is your yearly household income? (percentage, absolute number) |  |  |

**Table S2.** Proposed joint display for mixed methods integration

| Theme/ Measure | Int vs Ctrl | Pre-Post | Pre-quotes | Post-quotes | Integration |
| --- | --- | --- | --- | --- | --- |
| Isolation UCLA V3 |  |  |  |  |  |
| Depression  CES-D |  |  |  |  |  |
| Computer use CPQ-12 |  |  |  |  |  |
| Quality of life OPQOL-B |  |  |  |  |  |

Int = intervention group; Ctrl = control group

**Table S3**. Proposed feasibility outcomes

|  | Intervention (mean, SD) | Control (mean, SD) |
| --- | --- | --- |
| Number of participants enrolled |  |  |
| Adherence to the enTECH programming |  |  |
| Frequency of contact with the family |  |  |
| Rate of attrition |  |  |
| Challenges faced by volunteers |  |  |
